# Intraindividual double burden of overweight or obesity and micronutrient deficiencies or anemia among women of reproductive age in 17 population-based surveys

**DOI:** 10.1093/ajcn/nqaa118

**Published:** 2020-08-04

**Authors:** Anne M Williams, Junjie Guo, O Yaw Addo, Sanober Ismaily, Sorrel M L Namaste, Brietta M Oaks, Fabian Rohner, Parminder S Suchdev, Melissa F Young, Rafael Flores-Ayala, Reina Engle-Stone

**Affiliations:** Hubert Department of Global Health, Emory University, Atlanta, GA, USA; McKing Consulting Corporation, Atlanta, GA, USA; Hubert Department of Global Health, Emory University, Atlanta, GA, USA; Hubert Department of Global Health, Emory University, Atlanta, GA, USA; McKing Consulting Corporation, Atlanta, GA, USA; Hubert Department of Global Health, Emory University, Atlanta, GA, USA; The DHS Program, ICF International, Rockville, MD, USA; Department of Nutrition and Food Sciences, University of Rhode Island, Kingston, RI, USA; GroundWork, Fläsch, Switzerland; Department of Pediatrics, Emory University, Atlanta, GA, USA; Emory Global Health Institute, Atlanta, GA, USA; Division of Nutrition, Physical Activity and Obesity, US CDC, Atlanta, GA, USA; Hubert Department of Global Health, Emory University, Atlanta, GA, USA; Division of Nutrition, Physical Activity and Obesity, US CDC, Atlanta, GA, USA; Department of Nutrition, University of California, Davis, CA, USA

**Keywords:** double burden of malnutrition, women, anemia, overweight/obesity, micronutrients

## Abstract

**Background:**

Rising prevalence of overweight/obesity (OWOB) alongside persistent micronutrient deficiencies suggests many women face concomitant OWOB and undernutrition.

**Objectives:**

We aimed to *1*) describe the prevalence of the double burden of malnutrition (DBM) among nonpregnant women of reproductive age, defined as intraindividual OWOB and either ≥1 micronutrient deficiency [micronutrient deficiency index (MDI) > 0; DBM-MDI] or anemia (DBM-anemia); *2*) test whether the components of the DBM were independent; and *3*) identify factors associated with DBM-MDI and DBM-anemia.

**Methods:**

With data from 17 national surveys spanning low- and middle-income countries (LMICs) and high-income countries from the Biomarkers Reflecting Inflammation and Nutritional Determinants of Anemia project (*n* = 419 to *n* = 9029), we tested independence of over- and undernutrition using the Rao–Scott chi-square test and examined predictors of the DBM and its components using logistic regression for each survey.

**Results:**

Median DBM-MDI was 21.9% (range: 1.6%–39.2%); median DBM-anemia was 8.6% (range: 1.0%–18.6%). OWOB and micronutrient deficiencies or anemia were independent in most surveys. Where associations existed, OWOB was negatively associated with micronutrient deficiencies and anemia in LMICs. In 1 high-income country, OWOB women were more likely to experience micronutrient deficiencies and anemia. Age was consistently positively associated with OWOB and the DBM, whereas the associations with other sociodemographic characteristics varied. Higher socioeconomic status tended to be positively associated with OWOB and the DBM in LMICs, whereas in higher-income countries the association was reversed.

**Conclusions:**

The independence of OWOB and micronutrient deficiencies or anemia within individuals suggests that these forms of over- and undernutrition may have unique etiologies. Decision-makers should still consider the prevalence, consequences, and etiology of the individual components of the DBM as programs move towards double-duty interventions aimed at addressing OWOB and undernutrition simultaneously.

## Introduction

Until recently, efforts in low- and middle-income countries (LMICs) to improve the nutrition of women of reproductive age (WRA) largely focused on undernutrition (1). In the last decade, evidence has emerged showing that overweight/obesity (OWOB) prevalence now exceeds that of underweight among WRA in most LMICs (2, 3). In contrast to the declining prevalence of underweight among WRA (4), reductions in anemia prevalence, which is often used as a proxy for micronutrient deficiencies in the absence of micronutrient data (5), have been disappointing (6). Nearly all countries are off course to meet the World Health Assembly targets to reduce anemia among WRA by 50% between 2016 and 2025 (6); anemia prevalence decreased from 43% to 38% between 1995 and 2011 among nonpregnant WRA (7). As countries experience increases in OWOB alongside modest reductions of anemia, many face what has been termed the double burden of malnutrition (DBM) (8). The WHO describes the DBM as the “coexistence of overweight, obesity, or diet-related noncommunicable diseases with underweight or micronutrient deficiencies at the population, household, or individual level” (9). Failure to address the DBM is likely to have serious health and economic consequences for WRA and their nations (10–12), yet few LMICs have integrated OWOB reduction among WRA into national nutrition policies (13).

Country governments increasingly recognize the need to better target and design programs that address the full spectrum of malnutrition, and nutrition policies (e.g., supporting breastfeeding, and nutrition education) have the potential to simultaneously address over- and undernutrition. However, there are limited data on the magnitude and correlates of the DBM to inform double-duty actions, which are aimed at dually addressing OWOB and undernutrition (9,14). The breadth of operational definitions for the DBM further complicates the design of interventions to reduce the DBM. Although global estimates of obesity (15%), underweight (10%), and anemia (29%) among WRA have been characterized (15,16), a data gap remains for the burden of many micronutrient deficiencies and an even greater gap on the coexistence of multiple forms of malnutrition (4).

The ratio of obesity prevalence to undernutrition at the country level (17) or household co-occurrence of adult overnutrition and child undernutrition (18) are more common estimates of the DBM than at the intraindividual level among existing studies. As such, the extent to which over- and undernutrition are present in the same individual across different settings is unknown. One review in Latin America found the co-occurrence of overweight and anemia within WRA ranging from 3% to 14% among 5 countries (19). Data from the USA found obese and underweight women were at higher risk of vitamin deficiencies or anemia than normal-weight women (20). In Vietnam, weight category and micronutrient deficiencies were generally unrelated except that overweight women had better vitamin A status (21).

Leveraging data from population-based surveys of micronutrient status, our objectives were to *1*) describe the prevalence of the DBM among nonpregnant WRA, defined as intraindividual OWOB and either micronutrient deficiency index (MDI)  > 0 (DBM-MDI; OWOB and ≥1 micronutrient deficiency) or anemia (DBM-anemia); *2*) test whether the components of the DBM, using a range of definitions, were independent; and *3*) identify factors associated with DBM-MDI and DBM-anemia among WRA to guide intervention targeting. We focused our evaluation on DBM-MDI because few studies have used micronutrient deficiencies to characterize the undernutrition component of the DBM, and on DBM-anemia because anemia is the global target for women's nutrition (22).

## Methods

### Data source and inclusion criteria

The Biomarkers Reflecting Inflammation and Nutritional Determinants of Anemia (BRINDA) project harmonized individual participant data from multiple national nutrition surveys, which included data on anthropometry, anemia, inflammation, and micronutrient deficiencies (www.brinda-nutrition.org) (23). Methods describing the BRINDA database are available elsewhere (24), and survey reports or publications from these national surveys are available on the project website and in **Supplemental Table 1**. Briefly, to be included, surveys must have utilized a population-based representative sampling design, and measured hemoglobin or a biomarker of micronutrient status along with a biomarker of inflammation [C-reactive protein (CRP) or α-1-acid glycoprotein (AGP)]. Seventeen of 19 national surveys sampling WRA had anthropometry data. The inclusion criteria for this analysis were observations with nonmissing BMI (in kg/m^2^) and hemoglobin, ≥1 micronutrient biomarker [ferritin, soluble transferrin receptor (sTfR), retinol-binding protein (RBP), retinol, zinc, vitamin B-12, folate, or vitamin D], and a measure of inflammation (CRP or AGP), which resulted in a loss of 0%–1.2% of survey sample size. Height and weight outside the ranges 101.6–219.9 cm and 22.7–222.2 kg, respectively, were set to missing, as were BMI *z* scores >+5 and <−5, which accounted for 39 lost observations. All micronutrient biomarker values were retained with the exception of 1 apparent outlier (AGP > 500 g/L) and 26 hemoglobin concentrations outside the 40–180 g/L range, which brought the analytic sample to *n* = 419 to *n* = 9029 per survey. Hemoglobin was adjusted for altitude and smoking (25), where available (**Supplemental Table 2**).

### Creating the MDI

To consolidate information from the multiple micronutrient biomarkers available per survey, we developed an MDI to summarize the number of micronutrients for which biomarker concentrations indicated low status at the individual level. The MDI score ranged from 0, indicating no micronutrient deficiencies, to 6, the maximum number of micronutrients assessed in an individual survey. Cutoffs used to define deficiency were inflammation-adjusted ferritin < 15 μg/L (26, 27) or inflammation-adjusted sTfR > 8.3 mg/L (28), retinol or RBP < 0.7 μmol/L (29), vitamin B-12 < 150 pmol/L (30), serum folate < 10 nmol/L (31), and 25-hydroxyvitamin D < 30 nmol/L (32). Zinc cutoffs were <70 μg/dL (morning fasted), <66 μg/dL (morning nonfasted), and <59 μg/dL (afternoon) per International Zinc Nutrition Consultative Group recommendations (33). Supplemental Table 2 presents the methodologies for biomarker assessment by survey. The ordinal MDI score (range: 0–6) was collapsed into 3 levels with MDI = 0, MDI = 1, or MDI > 1, representing 0, 1, or multiple micronutrient deficiencies. We present results incorporating the MDI separately for surveys that collected 1–2 micronutrients, to prevent skewing results based on unavailable data.

### Defining OWOB and the DBM

The definition for OWOB depended on age category. For adolescent WRA, aged 15–19 y, we used the BMI-for-age *z* scores from the WHO growth reference data (34). Overweight ranged from +1 SD to +2 SD, and obesity was defined as BMI >+2 SD. For adult WRA > 19 y old, overweight and obesity were defined as BMI = 25 to <30 and BMI ≥ 30, respectively (35). Although OWOB cutoffs vary regionally, we adopted WHO cutoffs for consistency and because they are more conservative than cutoffs with lower bounds for our primary analyses (21). In secondary analyses, we also defined OWOB in Vietnam, Cambodia, and Laos as BMI > 23 (36). Underweight was defined as BMI-for-age *z* scores <−2 SD for adolescents and BMI < 18.5 for adults (35).

We initially defined the DBM 8 ways, each described with a suffix. Our primary focus was on intraindividual concomitant OWOB and MDI > 0 (DBM-MDI). An alternate definition was concomitant OWOB and anemia (hemoglobin < 12.0 g/dL; DBM-anemia). Concomitant OWOB and single micronutrient deficiencies were also evaluated: DBM-iron, DBM-vitamin A, DBM-zinc, DBM-vitamin B-12, DBM-folate, and DBM-vitamin D. In secondary analysis, overlapping forms of undernutrition were also described (short stature, defined as height < 145 cm, or underweight and micronutrient deficiencies or anemia).

### Variable definitions

Age categories were defined as 15–19, 20–29, 30–39, and 40–49 y old. The majority of surveys had an ordinal 3-level socioeconomic status (SES) variable derived from individual survey asset scores of household ownership or composition. In the USA, Georgia, and Papua New Guinea (PNG), the poverty-index ratio, employment (binary), and household income were used to create SES, respectively. A binary SES variable in Georgia was created for low SES (unemployment) or medium SES (any employment). Respondent (or household head: Mexico, 2006) education was collapsed into 2 levels: none or primary compared with secondary or higher. The 16 countries were grouped into 4 geographic areas based on the WHO regions to describe patterns of association: Americas (Mexico, Ecuador, USA, Colombia); Europe/Eastern Mediterranean (Azerbaijan, United Kingdom, Georgia, Afghanistan, Pakistan); Africa (Cameroon, Côte d'Ivoire, Malawi); and Southeast Asia/Western Pacific (PNG, Cambodia, Laos, Vietnam).

### Statistical analysis

Analyses were conducted in SAS version 9.4 (SAS Institute) separately for each survey with cluster, strata, and weights. Analyses were completed by 2 independent analysts; discrepancies were resolved through discussion and consensus. Descriptive characteristics and prevalence estimates were calculated using the SURVEYFREQ and SURVEYMEANS procedures. To test the independence of micronutrient deficiencies or anemia and OWOB, we compared observed and expected prevalence estimates of the DBM using the Rao–Scott chi-square test. The observed and expected DBM prevalence estimates excluded underweight women because they may be more likely to experience micronutrient deficiencies and anemia, creating a U-shaped relation between weight category and MDI > 0 or anemia. Our primary focus was micronutrient malnutrition rather than underweight and we wanted to narrow the comparison, to compare women with normal and elevated BMI. To model multivariable associations between sociodemographic characteristics and the DBM, the SURVEYLOGISTIC procedure was used (including the Firth option for zero observation cells). Multivariable models included age, SES, residence (urban/rural), and education based on data availability in each country. We also modeled multivariable associations between sociodemographic characteristics (age, SES, residence, education) and OWOB, MDI > 0, and anemia to better interpret DBM predictors.

### Ethical approval and role of the funding source

The study was reviewed by the institutional review board of the NIH (protocol #11417) and deemed non–human subjects research.

## Results

### Participant characteristics and prevalence of OWOB, micronutrient deficiencies, and anemia

Of the 17 nationally representative surveys in the analysis, Cambodia, Mexico 2012, and Pakistan did not sample women aged <20 y; all other surveys included nonpregnant women aged 15–49 y (**Table 1**). Rural residency ranged from 22.2% (Colombia) to 91.0% (Malawi), and education patterns varied from 100.0% of women in the USA reporting secondary/higher education to 84.4% of women in Côte d'Ivoire reporting none/primary education.

**TABLE 1:** Age, household characteristics, and educational attainment of women of reproductive age by survey: Biomarkers Reflecting Inflammation and Nutritional Determinants of Anemia project^1^

Geographic grouping	Country, survey year	*n*	Age,^2^ y	Rural residence	Low SES	Low education^3^
Americas	Mexico, 2012	3586	34.6 [22.3–41.1]	27.4 (25.7, 29.2)	38.3 (36.0, 40.7)	—
	Mexico, 2006	3006	31.1 [22.9–39.9]	29.6 (25.1, 34.0)	47.3 (43.3, 51.4)	59.9 (55.2, 62.6)
	Ecuador, 2012	7129	29.3 [21.4–37.9]	28.9 (18.6, 39.1)	38.9 (33.6, 44.3)	30.6 (27.8, 33.5)
	USA, 2006	3150	34.5 [24.4–42.3]	—	26.1 (23.0, 29.3)	0.0 (0.0, 0.0)
	Colombia, 2010	8809	27.6 [18.5–38.6]	22.2 (21.2, 23.2)	38.1 (36.7, 39.5)	51.1 (49.0, 53.2)
Europe/Eastern Mediterranean	Azerbaijan, 2013	2642	31.0 [23.4–41.1]	53.8 (47.6, 60.1)	31.3 (27.8, 34.8)	5.1 (3.6, 6.7)
	United Kingdom, 2014	876	35.7 [25.7–42.8]	—	36.1 (31.4, 40.9)	8.8 (6.1, 11.5)
	Georgia, 2009	1671	29.2 [26.5–32.0]	49.7 (42.8, 56.6)	19.3 (15.7, 22.8)	4.7 (3.2, 6.2)
	Afghanistan, 2013	568	29.0 [24.1–34.3]	—	7.2 (3.6, 10.9)	—
	Pakistan, 2011	9029	29.7 [25.7–34.6]	68.9 (66.1, 71.7)	42.5 (40.2, 44.8)	71.2 (69.3, 73.0)
Africa	Cameroon, 2009	748	26.0 [22.0–31.0]	41.5 (31.5, 51.6)	42.6 (35.3, 50.0)	66.4 (62.5, 70.3)
	Côte d'Ivoire, 2007	816	26.1 [20.9–32.0]	46.8 (42.9, 50.8)	38.8 (32.7, 44.9)	84.4 (80.8, 88.1)
	Malawi, 2016	758	25.3 [19.6–35.5]	91.0 (83.5, 98.4)	42.6 (35.2, 50.1)	79.6 (73.8, 85.5)
Southeast Asia/Western Pacific	Papua New Guinea, 2005	738	27.7 [20.7–35.3]	79.1 (69.3, 88.9)	39.5 (27.7, 51.3)	72.0 (66.6, 77.4)
	Cambodia, 2014	419	29.8 [25.1–33.3]	86.9 (83.6, 90.2)	43.7 (35.6, 51.7)	69.0 (64.1, 73.9)
	Laos, 2006	810	28.3 [20.0–36.8]	68.0 (54.2, 81.7)	38.8 (28.3, 49.4)	65.0 (55.3, 74.7)
	Vietnam, 2010	1480	31.9 [24.1–40.1]	51.4 (49.5, 53.2)	—	—

1 Values represent percentages (95% CIs) unless otherwise indicated; estimates account for cluster, strata, and weight. —, variable (or category) unavailable. SES, socioeconomic status.

2 Age shown as medians [IQRs].

3 Education categorized as binary for modeling purposes: low represents none or primary attained, higher education (secondary or beyond) not shown.

**FIGURE 1:**
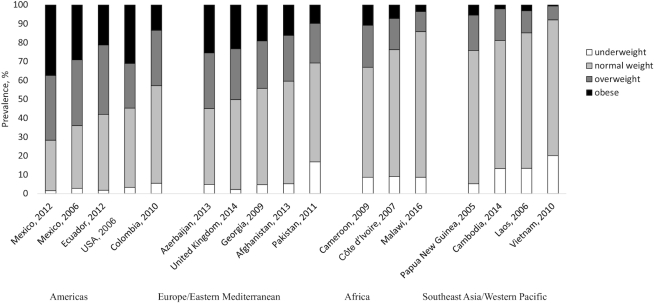
Distribution of BMI categories among women of reproductive age, by survey: Biomarkers Reflecting Inflammation and Nutritional Determinants of Anemia project. Anthropometry prevalence estimates are ordered from greatest overweight or obesity to lowest within geographic group. Estimates account for survey design (cluster, strata, weight). Definitions differ by age: BMI-for-age *z* scores were used for adolescents aged 15.0–19.0 y. Underweight was defined as BMI (in kg/m^2^) < 18.5 or BMI-for-age *z* score < −1; normal weight was defined as BMI = 18.5–24.9 or −1 ≤ BMI-for-age *z* score ≤ +1; overweight was defined as BMI = 25.0–29.9 or +1 < BMI-for-age *z* score ≤ +2; and obesity was defined as BMI ≥ 30 or BMI-for-age *z* score > +2.

The median prevalence of OWOB was 40.5% (range: 8.0%, Vietnam to 71.7%, Mexico 2012) (**Figure 1**). More than half of WRA were OWOB in 6 surveys (Mexico 2006/2012, Ecuador, USA, United Kingdom, and Azerbaijan). The prevalence of underweight was greatest in Southeast Asian countries (Cambodia, 13.3%; Laos, 13.4%; and Vietnam, 20.1%) and Pakistan (16.8%). Short stature ranged from <0.1% to 13.7% (**Supplemental Table 3**). Aside from Vietnam, all surveys had a greater proportion of OWOB than underweight women. When the population-specific definition for OWOB was applied to Cambodia, Laos, and Vietnam, the prevalence of OWOB increased by 15.7, 13.0, and 11.9 percentage points (pp), respectively.

Aside from iron status, which was assessed in all surveys, the number and pattern of micronutrients measured by survey varied. Individual micronutrient deficiency prevalence estimates ranged from 0.3% to 39.9% (vitamin A), 6.4% to 84.7% (zinc), 1.0% to 52.6% (vitamin B-12), 1.3% to 86.4% (folate), and 4.8% to 84.7% (vitamin D) (**Table 2**). Among 3 surveys that collected data on all 6 micronutrients, the prevalence of MDI = 0 (i.e., individuals with adequate status for all micronutrients) ranged from 8.0% (Pakistan) to 29.8% (Cambodia), and the prevalence of MDI > 1 ranged from 17.1% (Cambodia) to 72.1% (Pakistan) (Table 2). There were 5 surveys that measured 5 micronutrients, although the micronutrients measured varied. Among those surveys, the prevalence of MDI = 0 ranged from 12.3% to 66.3%. Among surveys that measured 1–2 micronutrients, the MDI = 0 prevalence ranged from 55.4% to 92.1% (Table 2).

**TABLE 2:** Prevalence of micronutrient deficiencies and anemia among women of reproductive age by survey: Biomarkers Reflecting Inflammation and Nutritional Determinants of Anemia project^1^

		MDI, %				Iron deficiency anemia	Prevalence of individual micronutrient deficiency, %					
Country, survey year	# MN	0	1	>1	Anemia		Iron	Vitamin A	Zinc	Vitamin B-12	Folate	Vitamin D
Pakistan, 2011	6	8.0 (7.1, 9.0)	19.9 (18.6, 21.1)	72.1 (70.4, 73.8)	50.6 (49.3, 51.8)	27.4 (26.2, 28.7)	43.2 (41.7, 44.7)	39.9 (37.7, 42.2)	53.3 (51.3, 55.3)	52.6 (50.4, 54.7)	54.1 (52.0, 56.3)	39.7 (37.6, 41.8)
Vietnam,^2^ 2010	6	22.2 (19.1, 25.2)	53.6 (50.8, 56.5)	24.2 (21.7, 26.7)	11.5 (9.3, 13.6)	5.8 (4.4, 7.2)	18.0 (15.7, 20.3)	1.3 (0.7, 1.9)	66.4 (62.4, 70.4)	12.1 (9.2, 14.9)	11.6 (9.7, 13.5)	17.6 (13.9, 21.3)
Cambodia, 2014	6	29.8 (22.2, 37.5)	53.1 (45.7, 60.5)	17.1 (12.7, 21.5)	44.1 (39.6, 48.5)	3.0 (0.8, 5.2)	3.5 (1.3, 5.7)	3.1 (1.4, 4.7)	60.7 (53.0, 68.3)	1.0 (0.00, 1.9)	17.8 (12.8, 22.8)	4.8 (2.1, 7.5)
Cameroon,^2^ 2009	5	12.3 (9.2, 15.4)	61.6 (57.4, 65.9)	26.0 (22.3, 29.8)	35.7 (31.0, 40.4)	13.3 (10.2, 16.4)	19.5 (16.0, 22.9)	1.5 (0.6, 2.3)	84.7 (81.0, 88.5)	15.4 (9.4, 21.4)	15.8 (11.0, 20.6)	—
Malawi, 2016	5	23.6 (18.4, 28.8)	47.0 (41.6, 52.3)	29.4 (25.7, 33.2)	22.2 (18.6, 25.8)	8.1 (5.6, 10.6)	15.1 (11.6, 18.7)	3.0 (1.3, 4.6)	62.7 (55.7, 69.6)	13.1 (9.1, 17.2)	19.2 (14.2, 24.3)	—
Ecuador,^2^ 2012	5	36.9 (34.7, 39.1)	49.2 (47.2, 51.1)	14.0 (12.9, 15.1)	14.6 (13.1, 16.0)	9.1 (7.7, 10.4)	18.7 (16.7, 20.7)	2.5 (1.4, 3.7)	57.1 (54.5, 59.6)	1.4 (1.0, 1.9)	1.3 (0.8, 1.8)	—
United Kingdom, 2014	5	50.9 (46.1, 55.7)	34.7 (30.3, 39.0)	14.4 (11.1, 17.8)	11.0 (8.1, 13.9)	5.6 (3.7, 7.5)	22.0 (17.6, 26.4)	1.2 (0.01, 2.5)	6.4 (4.5, 8.4)	8.1 (5.5, 10.6)	—	30.2 (25.7, 34.7)
USA, 2006	5	66.3 (63.4, 69.1)	27.9 (25.7, 30.1)	5.8 (4.6, 7.1)	6.6 (5.5, 7.7)	5.1 (4.2, 6.0)	20.6 (18.5, 22.6)	0.3 (0.05, 0.6)	—	2.8 (1.9, 3.8)	2.7 (1.8, 3.6)	13.8 (10.9, 16.7)
Afghanistan, 2013	4	7.1 (4.6, 9.7)	38.9 (33.1, 44.6)	54.0 (48.6, 59.5)	42.4 (37.2, 47.7)	16.7 (12.2, 21.1)	31.2 (24.1, 38.3)	11.4 (7.7, 15.1)	33.6 (25.9, 41.3)	—	—	84.7 (80.7, 88.8)
Côte d'Ivoire,^2^ 2007	4	14.6 (11.4, 17.8)	60.2 (56.0, 64.4)	25.2 (21.2, 29.2)	50.3 (46.7, 53.9)	15.2 (13.0, 17.4)	22.7 (19.4, 26.0)	0.7 (0.2, 1.3)	—	17.9 (11.0, 24.7)	86.4 (83.3, 89.6)	—
Azerbaijan,^2^ 2013	4	36.3 (33.2, 39.3)	42.4 (40.0, 44.8)	20.9 (18.2, 23.5)	38.1 (35.6, 40.7)	26.3 (24.2, 28.4)	43.2 (40.6, 45.8)	0.4 (0.1, 0.7)	—	19.5 (15.5, 23.5)	35.0 (31.3, 38.7)	—
Mexico, 2012	3	55.0 (52.8, 57.1)	42.0 (39.9, 44.1)	3.0 (2.4, 3.7)	13.5 (12.0, 14.9)	9.8 (8.5, 11.0)	43.7 (41.6, 45.8)	—	—	2.1 (1.6, 2.6)	2.8 (2.1, 3.4)	—
Mexico,^2^ 2006	2	55.4 (52.0, 58.9)	39.8 (36.5, 43.0)	4.8 (3.4, 6.1)	13.7 (11.4, 16.0)	8.6 (6.8, 10.3)	34.2 (31.2, 37.3)	—	24.4 (20.1, 28.7)	—	—	—
Georgia,^2^ 2009	2	79.8 (76.8, 82.7)	19.9 (17.0, 22.9)	0.3 (0.02, 0.6)	23.5 (20.4, 26.6)	0.7 (0.2, 1.2)	1.4 (0.8, 2.1)	—	—	—	80.3 (75.0, 85.6)	—
PNG, 2005	2	92.1 (89.8, 94.4)	7.6 (5.4, 9.9)	0.2 (0.0, 0.5)	35.8 (30.9, 40.7)	7.2 (4.9, 9.4)	7.6 (5.3, 9.9)	0.6 (0.0, 1.1)	—	—	—	—
Laos, 2006	1	73.6 (67.7, 79.6)	26.4 (20.4, 32.3)	—	35.9 (30.1, 41.7)	16.2 (11.8, 20.6)	26.4 (20.4, 32.3)	—	—	—	—	—
Colombia, 2010	1	74.4 (73.2, 75.6)	25.6 (24.4, 26.8)	—	7.5 (6.8, 8.3)	4.5 (4.0, 5.1)	25.6 (24.4, 26.8)	—	—	—	—	—

1 Values are percentages (95% CIs) unless started otherwise. Surveys in descending order of # MN and ascending order of MDI = 0. Estimates account for cluster, strata, and weight. Cutoffs to define deficiency: iron (inflammation-adjusted ferritin < 15 μg/L, inflammation-adjusted soluble transferrin receptor > 8.3 mg/L for PNG); vitamin A (retinol-binding protein or retinol < 0.7 μmol/L); serum zinc according to the International Zinc Nutrition Consultative Group (taking into account fasting and time of collection, when available); vitamin B-12 < 150 pmol/L; serum folate < 10 nmol/L (RIA Bio-Rad assay) or <6.8 nmol/L (microbiologic assay); 25-hydroxyvitamin D < 30 nmol/L; and anemia (hemoglobin adjusted for smoking and altitude < 12.0 g/dL). MDI is the sum of biomarkers with values below the thresholds that define deficiency. MDI, Micronutrient Deficiency Index; PNG, Papua New Guinea; # MN, micronutrients measured.

2 Surveys that subsampled select micronutrients are Ecuador (75%, vitamin A); Azerbaijan, Cameroon, Côte d'Ivoire, and Vietnam (30–50%, vitamin B-12); Cameroon (50%, folate); Mexico, 2006 (60%, zinc); and Georgia (20%, folate).

Anemia prevalence ranged from 6.6% (USA) to 50.6% (Pakistan) (Table 2). Based on the WHO criteria of anemia severity (37), the public health problem was severe (anemia ≥ 40%) in 4 surveys, moderate (anemia = 20.0%–39.9%) in 6 surveys, and mild (anemia = 5.0%–19.9%) in 7 surveys. The prevalence of inflammation-adjusted ferritin < 15 μg/L ranged from 1.4% (Georgia) to 43.7% (Mexico 2012). Iron deficiency anemia prevalence ranged from 0.7% (Georgia) to 27.4% (Pakistan) (Table 2).

### Prevalence of the DBM

Among the 12 surveys that collected information on ≥3 micronutrients, the prevalence of DBM-MDI ranged from 7.5% (Vietnam) to 39.2% (Afghanistan) (**Table 3**) with a median prevalence of 23.4%. In 5 surveys that collected information on only 1–2 micronutrients, the prevalence of DBM-MDI ranged from 1.6% (PNG) to 28.8% (Mexico 2006) with a median prevalence of 10.4% (Table 3). Prevalence of DBM-anemia ranged from 1.0% (Vietnam) to 18.6% (Afghanistan) with a median prevalence of 8.6% (Table 3). Using a population-specific definition of OWOB for Cambodia, Laos, and Vietnam, the prevalence of DBM-MDI and DBM-anemia increased by a mean 7.5 pp and 3.3 pp, respectively. Prevalence estimates ranged for concomitant OWOB and single micronutrient deficiencies: DMB-iron, 0.0% (Cambodia) to 31.3% (Mexico 2012); DBM-vitamin A, 0.0% (PNG, Cameroon, Azerbaijan) to 13.4% (Pakistan); DBM-zinc, 3.2% (United Kingdom) to 33.6% (Ecuador); DBM-vitamin B-12, 0.0% (Cambodia) to 20.6% (Pakistan); DBM-folate, 0.8% (Ecuador) to 40.9% (Georgia); and DBM-vitamin D, 0.5% (Cambodia) to 35.3% (Afghanistan) (Table 3).

**TABLE 3:** Prevalence estimates of the percentage of concomitant OWOB and micronutrient deficiencies or anemia among women of reproductive age with BMI > 18.5 kg/m^2^ by survey: Biomarkers Reflecting Inflammation and Nutritional Determinants of Anemia project^1^

Geographic grouping	Country, survey year	Anemia	MDI^2^ > 0	Iron	Vitamin A	Zinc	Vitamin B-12	Folate	Vitamin D
		Obs.	Obs.	Obs.	Obs.	Obs.	Obs.	Obs.	Obs.
Americas	Mexico, 2012	9.9	32.3	31.3	—	—	1.5	1.9	—
	Mexico,^3^ 2006	9.0	28.8	22.3	—	15.4	—	—	—
	Ecuador,^3^ 2012	8.5	35.6*↓	9.5***↓	1.5	33.6	0.9	0.8	—
	USA, 2006	4.6**↑	21.9***↑	12.4	0.2	—	1.6	2.1**↑	10.5***↑
	Colombia, 2010	3.5	10.9*↓	10.9*↓	—	—	—	—	—
Europe/Eastern Mediterranean	Azerbaijan,^3^ 2013	8.7	17.7	11.0	0.0	—	8.2	8.4	—
	United Kingdom, 2014	4.8	26.1	10.3	0.9	3.2	4.8	—	18.5**↑
	Georgia,^3^ 2009	10.3	10.4	0.7	—	—	—	40.9	—
	Afghanistan, 2013	18.6	39.2	14.7	4.7	14.1	—	—	35.3
	Pakistan, 2011	15.4***↓	34.0	14.8*↓	13.4*↓	18.2***↓	20.6**↑	19.8	16.1**↑
Africa	Cameroon,^3^ 2009	8.9***↓	30.7	7.5	0.0	29.4	4.1	7.6	—
	Côte d'Ivoire,^3^ 2007	11.9	23.4	6.6	0.1	—	2.4**↓	24.4***↑	—
	Malawi, 2016	3.6	12.2	2.9	0.1	11.0	1.0**↓	3.8	—
Southeast Asia/Western Pacific	PNG, 2005	5.3**↓	1.6	2.8**↓	0.0	—	—	—	—
	Laos, 2006	4.8	2.3**↓	2.3**↓	—	—	—	—	—
	Cambodia, 2014	8.6	13.1	0.0	1.0	11.5	0.0	3.7	0.5
	Vietnam,^3^ 2010	1.0	7.5	1.5	0.2	6.4	1.5	1.1	1.2

1 Values are percentages. Surveys in descending order of OWOB prevalence within geographic groups. Differences between observed and expected prevalence estimates calculated using the Rao–Scott modified chi-square test accounting for complex survey design variables (cluster, strata, and weight). Women with BMI < 18.5 kg/m^2^ were removed from this analysis. Cutoffs to define deficiency: anemia (hemoglobin adjusted for smoking and altitude < 12.0 g/dL); iron (inflammation-adjusted ferritin < 15 μg/L or soluble transferrin receptor > 8.3 mg/L); vitamin A (retinol-binding protein or retinol < 0.7 μmol/L); zinc according to the International Zinc Nutrition Consultative Group; vitamin B-12 < 150 pmol/L; folate < 10 nmol/L (RIA Bio-Rad assay) or <6.8 nmol/L (microbiologic assay); and 25-hydroxyvitamin D < 30 nmol/L. ^*,**,***^Significance: *P < 0.05; **P < 0.01; ***P < 0.0001; ↑, observed prevalence was higher than expected; ↓, observed prevalence was lower than expected. —, micronutrient not measured. MDI, Micronutrient Deficiency Index; OWOB, overweight/obesity; PNG, Papua New Guinea.

2 Surveys that measured <3 micronutrients, and therefore have less opportunity to have a high prevalence of MDI > 0, were Mexico, 2006; Colombia, 2010; Georgia, 2009; PNG, 2005; and Laos, 2006.

3 Subsampled biomarkers and surveys include Mexico, 2006 (zinc, 60%); Ecuador (vitamin A, 75%); Azerbaijan (vitamin B-12, 50%); Georgia (folate, 20%); Cameroon (vitamin B-12, 50%; folate, 50%); Côte d'Ivoire (vitamin B-12, 50%); and Vietnam (vitamin B-12, 30%). Subsampling explains discrepancies between MDI > 0 and the individual micronutrient deficiencies (e.g., Georgia).

### Independence of OWOB and micronutrient deficiencies or anemia

In 13 of 17 surveys, the prevalence of DBM-MDI was no different than what would be expected by chance, assuming independent distributions of each condition (Table 3). In Colombia, Ecuador, and Laos, OWOB women were less likely to have MDI > 0 than normal-weight women (*P *< 0.02). In the USA, OWOB women were more likely to have MDI > 0 than normal-weight women (*P *< 0.0001) (Table 3). The patterns of independence were similar between DBM-MDI and DBM-anemia; in 13 of 17 surveys OWOB was independent of anemia (Table 3). DBM-anemia prevalence in Pakistan, Cameroon, and PNG was ∼3 pp lower than expected (*P *< 0.001), indicating lower odds of anemia among OWOB women. In the USA, OWOB women were more likely to have anemia than normal-weight women (*P* = 0.006) (Table 3). These associations persisted when controlling for age and were not affected when population-specific BMI cutoffs were used to define OWOB.

The patterns of independence between micronutrient deficiencies and OWOB differed by micronutrient, but overwhelmingly the 2 conditions were not associated. The exception was for vitamin D where in 3 of 6 surveys that measured vitamin D (Pakistan, United Kingdom, and USA), OWOB women were more likely to be vitamin D deficient than expected (Table 3). In 5 of 17 surveys, iron deficiency and OWOB were associated and, among these, the observed prevalence of DBM-iron was consistently less than expected; that is, individuals with OWOB were less likely to have iron deficiency than those with normal BMI. In 2 of 11 surveys that measured folate, a higher prevalence of DBM-folate than expected was observed in Côte d'Ivoire and the USA. There were significant associations of OWOB with vitamin B-12, vitamin A, and zinc deficiency in 3 of 11, 1 of 12, and 1 of 9 surveys, respectively (Table 3).

### Predictors of the DBM

**FIGURE 2:**
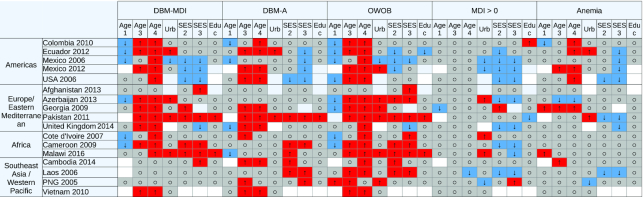
Patterns of associations between age, SES, residence, and education for OWOB, anemia, micronutrient deficiencies, and intraindividual DBM among women of reproductive age with BMI (in kg/m^2^) > 18.5 by survey, organized by geographic groupings: Biomarkers Reflecting Inflammation and Nutritional Determinants of Anemia project. Blue (**↓**) indicates protective, red (**↑**) indicates risk factor, gray (**○**) indicates no association, and white indicates the variable was unavailable. The column exposure variables represent the following: Age1 (15–19 y), Age2 (20–29 y; ref), Age3 (30–39 y), Age4 (40–49 y); Urb (urban; ref = rural); SES1 (low SES; ref), SES2 (medium SES), SES3 (high SES); Educ (secondary or higher education; ref = none or primary education). Women in the normal BMI category served as the reference and women with BMI < 18.5 were removed from these analyses. Supplemental Tables 4–8 present the adjusted ORs used to populate the figure. DBM, double burden of malnutrition; DBM-anemia, double burden of malnutrition, defined using anemia as the indicator of undernutrition; DBM-MDI, double burden of malnutrition, defined using micronutrient deficiency as the indicator of undernutrition; MDI, Micronutrient Deficiency Index; OWOB, overweight/obesity; PNG, Papua New Guinea; SES, socioeconomic status.

Age had the most consistent patterns of association with the DBM across surveys. Older age was associated with higher odds of DBM-MDI in 9 and 13 surveys for women aged 30–39 y and 40–49 y, respectively (**Figure 2**). Among these, the odds of DBM-MDI ranged from 1.5 (95% CI: 1.1, 2.2) (Cameroon) for 30–39 y to 3.5 (95% CI: 2.0, 6.1) (Vietnam) for 40–49 y, compared with 20–29 y (**Supplemental Table 4**). Patterns of association were similar between DBM-MDI and DBM-anemia with respect to age: younger women (15–19 y) had lower odds of DBM-MDI in 6 surveys and DBM-anemia in 4 surveys (remainder nonsignificant) (Figure 2, Supplemental Table 4, **Supplemental Table 5**). Older women (aged 30–39 y or 40–49 y) had higher odds of DBM-anemia in 11 surveys. The association between age and the DBM mirrored the association between age and OWOB. Being 15–19 y old was associated with lower odds of OWOB in 8 of 13 surveys, and being older was associated with higher odds of OWOB in 16 of 17 surveys (Figure 2, **Supplemental Table 6**).

In the Americas region, women with higher SES had lower or similar odds of DBM-MDI and DBM-anemia compared with women with lower SES. Higher SES was also associated with lower odds of DBM-MDI in the United Kingdom. In the Africa and Southeast Asia/Western Pacific geographic groupings, higher SES was associated with higher or similar odds of DBM-MDI and DBM-anemia (Figure 2, Supplemental Tables 4, 5). Higher SES was associated with higher odds of OWOB in 8 of 16 surveys, covering all geographic groups except the Americas. In contrast, higher SES was sporadically associated with lower odds of anemia (7 of 16 surveys) and MDI > 0 (9 of 16 surveys) (Figure 2, **Supplemental Tables 7**, **8**).

In most surveys, associations between DBM-MDI or DBM-anemia and urban residence or higher education were not significant in adjusted models, except for 2 (or 4) surveys with higher odds in urban residence for DBM-anemia (or DBM-MDI) and 1 (or 2) surveys with higher odds among higher-educated women for DBM-anemia (or DBM-MDI) (Figure 2, Supplemental Tables 4, 5). Urban residence and higher education were associated with greater odds of OWOB in 6 of 14 and 2 of 13 surveys, respectively. Urban residence was not often associated with anemia or MDI > 0 (in 3 of 14 and 6 of 14 surveys, respectively); yet, when there was an association it was more often with higher odds of anemia (Figure 2, Supplemental Tables 7, 8).

## Discussion

Intraindividual DBM was common among women from 17 nationally representative surveys in diverse geographic locations, affecting on average 1 in 4 women. However, the prevalence depended largely on how the DBM was defined. In all but 2 surveys, concomitant micronutrient deficiencies and OWOB was more prevalent than DBM-anemia. However, the conditions of over- and undernutrition were overwhelmingly independent. Where OWOB and undernutrition were associated with one another, a higher weight category tended to be associated with lower prevalence of anemia and micronutrient deficiencies, especially in LMICs. The independence of over- and undernutrition questions the practicality of a risk factor analysis for the DBM. We decided a priori to assess the correlates of the DBM along with its prevalence. In most countries, DBM correlates patterned after correlates of OWOB (although some discordance was observed). A synthesis of these findings suggests that over- and undernutrition do not necessarily need to be targeted simultaneously within individuals to successfully address the DBM.

Our study findings highlight the influence that the definition of the DBM has on its magnitude. When the DBM has been estimated at the household level with malnourished dyads (e.g., stunted child and OWOB mother), modest prevalence estimates ranged from <5% in a review of sub-Saharan Africa (18) to predominantly <10% (38,39). Intraindividual definitions of the DBM in women using OWOB and anemia were reported to realize prevalence estimates of ≤22% in urban strata (19,40). A DBM defined using cardiometabolic risk factors overlapping with nutritional deficiencies found 23.5% of adults in Burkina Faso affected, with higher prevalence among WRA (41). We found a median DBM-MDI prevalence of 21.9% (range: 1.6%–39.2%), or, in surveys measuring ≥3 micronutrients, 23.4% (range: 7.5%–39.2%), whereas the median DBM-anemia prevalence was 8.6% (range: 1.0%–18.6%). Median prevalence for DBM-iron was 10.3% (range: 0.0%–31.3%). Across surveys, DBM-iron prevalence ranged from 21.4 pp higher to 9.6 pp lower than DBM-anemia prevalence, which furthers the evidence that anemia may not be an appropriate proxy for iron deficiency in the absence of iron status data (5,42). Using the United Kingdom as an example, DBM-MDI was 26.1% whereas DBM-anemia was 4.8%, suggesting very different magnitudes of the DBM. Further investigation of the relative contributions of individual micronutrients to DBM-MDI within the United Kingdom suggests that vitamin D and iron are the micronutrients of greatest concern (DBM-vitamin D = 18.5%, DBM-iron = 10.3%), as well as OWOB. These various definitions influence the prevalence estimates of the DBM as well as their interpretation for program development.

As new indexes of micronutrient deficiencies are developed and applied, it is likely still useful to present results for individual micronutrients. In a few cases, the MDI masked associations between single micronutrient deficiencies and OWOB. For example, in Pakistan MDI > 0 and OWOB were not statistically associated, although OWOB Pakistani women were more likely to be deficient in vitamins B-12 and D and less likely to be deficient in iron, vitamin A, and zinc. Similarly, in Côte d'Ivoire, OWOB women were more likely to be folate deficient, less likely to be vitamin B-12 deficient, and there was no association between MDI > 0 and OWOB. Without assessing the relation between individual micronutrients and OWOB, this granularity would have been overlooked. Using multiple micronutrients for defining undernutrition is further convoluted by the distinct interaction between micronutrients and the proinflammatory condition of OWOB (27–29). For example, we did not find an increased risk of iron deficiency among OWOB women as was observed for obese women in Mexico and the USA (43,44). Five surveys exhibited an observed DBM-iron prevalence lower than expected (Ecuador, Colombia, Pakistan, PNG, and Laos) which contradicts OWOB populations being at higher risk of iron deficiency (45–47). Our findings were more consistent with a study in Nicaragua where OWOB women were less likely to have iron deficiency (48). Although the MDI was useful for consolidating micronutrient deficiencies, single micronutrient DBM estimates may be more informative for intervention development.

OWOB and micronutrient deficiencies or anemia were independent in the majority of surveys, but when associated OWOB women in LMICs were less likely to have anemia or micronutrient deficiencies. These patterns of association persisted when controlling for age and SES. Although the differences between observed and expected prevalence estimates tended to be small, they could translate to large discrepancies in micronutrient deficiencies or anemia by BMI category. In Laos, normal-weight women were approximately twice as likely to have micronutrient deficiencies as OWOB women, and in Cameroon 40% of normal-weight women had anemia compared with 25% of OWOB women (data not shown). The independence of over- and undernutrition has been described among stunted child/overweight mother pairs (49), and in India where 19 phenotypes of the DBM were described (50). Negative associations between measures of undernutrition (stunting, anemia) and obesity have also been reported (19), challenging the notion that “obesity is generally associated with worse micronutrient status” (51). Instead, these associations may indicate that OWOB women in certain settings are generally better-nourished than women with BMI between 18 and 25. OWOB may be reflective of prosperity and nutrient excess (macro- and micronutrient) in LMICs entering the nutrition transition. That can be contrasted with the positive association between OWOB and undernutrition in the USA. The single micronutrient analyses of independence from OWOB revealed an interesting pattern with vitamin D, where the most positive associations were found respective to how many comparisons were possible. OWOB women were more likely to be vitamin D deficient than normal-weight women in 3 of 6 surveys in which this analysis could be conducted. The pathophysiology of overweight or obesity on vitamin D status may be implicated in this finding (52, 53).

In general, we found similar age and SES patterns predicting DBM-anemia, DBM-MDI, and OWOB, suggesting that program targeting for the DBM would pattern after targeting for OWOB. Therefore, because OWOB individuals and their households are targeted in obesity prevention or treatment programs, program managers should be aware that many of these individuals are likely to have concomitant micronutrient deficiencies or anemia. This has been demonstrated at the household level (54), but is likely less well documented at the individual level. DBM-MDI and DBM-anemia were largely unassociated with urban residence (exceptions: Mexico 2006, Ecuador, Azerbaijan, Pakistan, Malawi), possibly owing to the trend of increasing OWOB in rural areas (3). Similarly, higher education was generally unassociated with DBM-MDI and DBM-anemia, but these findings must be interpreted with caution given that education was harmonized and may not mean the same thing across countries.

The number of harmonized national nutrition surveys available for analysis, and the investigation of the DBM using micronutrient deficiencies, were strengths of this analysis. However, the cross-sectional nature of the data is a limitation and precludes any life-course analysis of the DBM. We were further limited in the risk factors analysis based on data availability (e.g., parity was unavailable in most of the survey data sets). The surveys included in this study are a convenience sample of nationally representative surveys and therefore cannot answer questions for the DBM globally. Alternate risk factor analyses, such as examining the independence of OWOB and micronutrient deficiencies within different population subgroups (e.g., SES categories), could aid in the understanding of the risk factor patterns that were observed between the DBM and OWOB. Variation in the micronutrients measured across surveys and inconsistencies of field procedures and laboratory methods are additional limitations that we addressed by doing survey-specific analyses. The increased probability of MDI > 0 in surveys that measured more micronutrients is noteworthy, but we limited discussion of patterns of the MDI to among surveys that measured the same micronutrients. Another limitation is that certain micronutrient indicators (e.g., zinc and retinol/RBP) are intended for population assessment (29,33) but were used at the individual level. Data on dietary intake, physical activity, and cardiometabolic risk biomarkers would have been useful for the risk factor analysis. Nevertheless, these analyses characterize multiple definitions of intraindividual DBM and identify populations most affected. Future work is needed to understand contextualized situations within countries.

Although we are unable to ascribe causality with this study design, the limited and negative associations between OWOB and undernutrition that we and others have described at household and regional levels (54–56) suggest that OWOB and micronutrient deficiencies or anemia may have separate context-specific etiologies. Therefore, we urge program managers to not abandon interventions designed to address only 1 facet of the DBM. Micronutrient deficiencies ranged from <8% to >90% across surveys, highlighting that the micronutrients of greatest concern differ substantially by country. Program synergies may be explored, as suggested by recent calls for “double-duty” interventions (9,57), but rigorous testing of these interventions for the explicit purpose of reducing multiple forms of malnutrition is lacking. Understanding the effectiveness of single interventions that aim to simultaneously reduce over- and undernutrition along with interventions combatting components of the DBM could help with identifying the most appropriate intervention strategy. In addition, in settings where OWOB women are less likely to be anemic or micronutrient deficient, programs to address OWOB can incorporate careful planning and monitoring to ensure that micronutrient deficiencies or anemia are not exacerbated. For example, a poorly designed obesity prevention program that focuses on caloric reduction but does not meet individual micronutrient requirements may lead populations to reduce their intake of energy-dense nutrient-dense foods instead of energy-dense nutrient-poor foods. Similarly, programs targeting the reduction of micronutrient deficiencies or anemia among women need to ensure that OWOB prevalence does not rise as a result of the program.

Concomitant OWOB and micronutrient deficiencies affected >20% of WRA in the majority of countries we examined. The conditions of OWOB and micronutrient deficiencies or anemia were largely independent. These observations suggest that interventions to address the components of the DBM may still lead to reductions in the DBM, but double-duty interventions to address multiple facets of malnutrition simultaneously merit exploration and evaluation. Given the heterogeneity in prevalence and correlates of the DBM by survey, leveraging country-specific data will be a critical step in developing programmatic responses.

## Supplementary Material

nqaa118_Supplemental_File

## Notes

Supported by the Bill & Melinda Gates Foundation, CDC, Eunice Kennedy Shriver National Institute of Child Health and Human Development, HarvestPlus, and the United States Agency for International Development.

Supplemental Tables 1–8 are available from the “Supplementary data” link in the online posting of the article and from the same link in the online table of contents at https://academic.oup.com/ajcn/.

Data described in the article, code book, and analytic code will be made available upon request pending approval from the BRINDA steering committee and country representatives.

Publication costs for this supplement were defrayed in part by the payment of page charges by the Bill & Melinda Gates Foundation. The Guest Editor for this supplement was Daniel Roth, who has no relevant disclosures. The Supplement Coordinators for the supplement publication were Parminder S Suchdev and Melissa F Young, Emory University, Atlanta, GA 30322, USA. Supplement Coordinator disclosure: PSS receives salary support from the CDC; MFY: no conflicts to disclose. The opinions expressed in this publication are those of the authors and are not attributable to the sponsors or the publisher, Editor, or Editorial Board of *The American Journal of Clinical Nutrition*.

Abbreviations used: AGP, α-1-acid glycoprotein; BRINDA, Biomarkers Reflecting Inflammation and Nutritional Determinants of Anemia; CRP, C-reactive protein; DBM, double burden of malnutrition; DBM-anemia, double burden of malnutrition, defined using anemia as the indicator of undernutrition; DBM-MDI, double burden of malnutrition, defined using micronutrient deficiency as the indicator of undernutrition; LMIC, low- and middle-income country; MDI, Micronutrient Deficiency Index; OWOB, overweight/obesity; PNG, Papua New Guinea; pp, percentage points; RBP, retinol-binding protein; SES, socioeconomic status; sTfR, soluble transferrin receptor; WRA, women of reproductive age.
