# Supplementary material for: Intraindividual double burden of overweight or obesity and micronutrient deficiencies or anemia among women of reproductive age in 17 population-based surveys
Source: Am J Clin Nutr. 2020 Aug 4;112(Suppl 1):468S–477S. doi: 10.1093/ajcn/nqaa118 (PMC7396267; doi:10.1093/ajcn/nqaa118)
Supplement: nqaa118_Supplemental_File [file nqaa118_supplemental_file.docx]

**Supplemental material for Williams AM et al. Intra-individual double burden of overweight or obesity and micronutrient deficiencies or anemia among women of reproductive age in population-based surveys**

Supplemental Table 1. Survey report citations or publications (with hyperlinks) that correspond to the survey data compiled for analysis in the BRINDA project that were included in this study^1^

| Afghanistan, 2013 | UNICEF. 2014. [National nutrition survey: Afghanistan (2013)](http://nutritionmoph.gov.af/wp-content/uploads/2018/National%20Nutrition%20Survey/NNS%20-%202013%20Report.pdf). Kabul: United Nations Children’s Fund (UNICEF). |
| --- | --- |
| Azerbaijan, 2013 | UNICEF. 2013. [Azerbaijan nutrition survey (AzNS)](http://groundworkhealth.org/wp-content/uploads/2015/06/UNICEF-2013_Azerbaijan-National-Nutrition-Survey_report_eng_compressed.pdf) |
| Cambodia, 2014 | Wieringa FT, Dahl M, Chamnan C, Poirot E, Kuong K, Sophonneary P, et al. [The High Prevalence of Anemia in Cambodian Children and Women Cannot Be Satisfactorily Explained by Nutritional Deficiencies or Hemoglobin Disorders.](https://www.ncbi.nlm.nih.gov/pubmed/27338454) Nutrients. 2016;8(6). |
| Cameroon, 2009 | Engle-Stone R, Ndjebayi AO, Nankap M, Killilea DW, Brown KH. [Stunting prevalence, plasma zinc concentrations, and dietary zinc intakes in a nationally representative sample suggest a high risk of zinc deficiency among women and young children in Cameroon.](https://www.ncbi.nlm.nih.gov/pubmed/24453129) J Nutr. 2014;144(3):382-91. |
| Colombia, 2010 | de Lleras, C. D. L. F. 2010. [Encuesta Nacional de la situación nutricional en Colombia 2010](https://www.minsalud.gov.co/sites/RID/Lists/BibliotecaDigital/RIDE/VS/ED/GCFI/Base%20de%20datos%20ENSIN%20-%20Protocolo%20Ensin%202010.pdf) [National Survey of the nutritional situation in Colombia 2010]. |
| Côte d’Ivoire, 2007 | Rohner, F., Tschannen, A. B., Northrop-Clewes, C., Kouassi-Gohou, V., Bosso, P. E., & Mascie-Taylor, C. N. 2012. [Comparison of a possession score and a poverty index in predicting anaemia and undernutrition in pre-school children and women of reproductive age in rural and urban Cote d'Ivoire.](https://www.ncbi.nlm.nih.gov/pubmed/22687630) Public Health Nutrition, 15(9), 1620-1629. |
| Ecuador, 2012 | Freire, W. B., Belmont, P., López-Cevallos, D. F., & Waters, W. F. (2015). [Ecuador's National Health and Nutrition Survey: objectives, design, and methods.](https://www.ncbi.nlm.nih.gov/pubmed/26386743) Annals of epidemiology, 25(11), 877-878. |
| Georgia, 2009 | UNICEF. 2010. Report of the Georgia National Nutrition Survey, 2009. |
| Laos, 2006 | Laos Service national de la statistique, Laos Hygiene and Prevention Department. National Maternal and Child Nutrition Survey (MICS3-NNS) Report, the Lao PDR, 2006: Final Report: Ministry of Planning and Investment, Department of Statistics, 2009. |
| Malawi, 2016 | National Statistical Office (NSO), Community Health Sciences Unit (CHSU) [Malawi], Centers for Disease Control and Prevention (CDC), and Emory University. 2017. *[Malawi Micronutrient Survey 2015-16.](https://dhsprogram.com/pubs/pdf/FR319/FR319.m.final.pdf)* Atlanta, GA, USA: NSO, CHSU, CDC and Emory University. |
| Mexico, 2006 | Abúndez, C. O., Cázares, G. N., et al. 2006. [Encuesta nacional de salud y nutrición 2006](https://ensanut.insp.mx/encuestas/ensanut2006/doctos/informes/ensanut2006.pdf) [National Health and Nutrition Survey 2006]. Instituto Nacional de Salud Pública. |
| Mexico, 2012 | Gutierrez, J. P., Rivera-Dommarco, J., Shamah-Levy, T., Villalpando-Hernández, et al. 2012. [Encuesta nacional de salud y nutrición 2012](https://ensanut.insp.mx/encuestas/ensanut2012/doctos/informes/ENSANUT2012ResultadosNacionales2Ed.pdf) [National Health and Nutrition Survey 2012]. Resultados Nacionales. Cuernavaca, México: Instituto Nacional de Salud Pública, 1(1.48). |
| Pakistan, 2011 | Di Cesare M, Bhatti Z, Soofi SB, Fortunato L, Ezzati M, Bhutta ZA. [Geographical and socioeconomic inequalities in women and children's nutritional status in Pakistan in 2011: an analysis of data from a nationally representative survey](https://www.ncbi.nlm.nih.gov/pubmed/25794676). Lancet Glob Health. 2015;3(4):e229-39. |
| Papua New Guinea, 2005 | National Department of Health, U. P., University of Papua New Guinea, US Centre for Disease Control. 2011. [Papua New Guinea National Nutrition Survey, 2005 (PNG NNS 2005).](https://www.pacjmedsci.com/PJMS%20Vol%208%20No%202%20special%20issue%20May%202011.pdf) Pacific Journal of Medical Sciences Volume 8, No. 2 |
| United Kingdom, 2014 | Public Health England, Food Standards Agency, and MRC Human Nutrition. [National Diet and Nutrition Survey Results from Years 5 and 6 (combined) of the Rolling Programme (2012/13 – 2013/14).](https://assets.publishing.service.gov.uk/government/uploads/system/uploads/attachment_data/file/551352/NDNS_Y5_6_UK_Main_Text.pdf) |
| United States, 2006 | Centers for Disease Control and Prevention. 2012. [Second national report on biochemical indicators of diet and nutrition in the US population. Atlanta (GA): CDC](https://www.cdc.gov/nutritionreport/pdf/Nutrition_Book_complete508_final.pdf) |
| Vietnam, 2010 | Laillou, A., Van Pham, T., Tran, N. T., Le, H. T., Wieringa, F et al. 2012. [Micronutrient deficits are still public health issues among women and young children in Vietnam. PloS one, 7(4).](https://www.ncbi.nlm.nih.gov/pmc/articles/PMC3328495/) |

1. BRINDA: Biomarkers Reflecting Inflammation and Nutritional Determinants of Anemia

Supplemental Table 2. Biomarker availability and lab methods by survey^1^

| Country, survey year | Micronutrient biomarkers | | | | | | | | Hemoglobin adjusted for | | Hemocue model and blood collection technique |
| --- | --- | --- | --- | --- | --- | --- | --- | --- | --- | --- | --- |
|  | Iron | | Vitamin A | | Zinc | Vitamin B12 | Folate | Vitamin D | altitude | smoking |  |
|  | biomarker | method | biomarker | method |  |  |  |  |  |  |  |
| Afghanistan, 2013 | Ferritin |  | Retinol | UNK | AAS | -- | -- | HPLC | Yes | No | UNK |
| Azerbaijan, 2013 | Ferritin | ELISA | RBP | ELISA | -- | Microbiologic assay | Microbiologic assay | -- | Yes | Yes^2^ | ® 201^┼^ |
| Cambodia, 2014 | Ferritin | ELISA | RBP | UNK | AAS | Immunoassay | Immunoassay | ELISA | No | Yes | ® 301 |
| Cameroon, 2009 | Ferritin | ELISA | RBP | UNK | AAS | Radioassay | Radioassay | -- | No | No | ® 201^┼^ |
| Colombia, 2010 | Ferritin | UNK | -- | -- | -- | -- | -- | -- | Yes | Yes | Hb-B |
| Côte d’Ivoire, 2007 | Ferritin | ELISA | RBP | ELISA | -- | Microbiologic assay | Microbiologic assay | -- | No | No | ® 201^┼^ |
| Ecuador, 2012 | Ferritin | UNK | Retinol | UNK | AAS | Immunoassay | Immunoassay | -- | Yes | Yes | UNK |
| Georgia, 2009 | Ferritin | UNK | -- | -- | -- | -- | Microbiologic assay | -- | Yes | Yes | UNK |
| Laos, 2006 | Ferritin |  | -- | -- | -- | -- | -- | -- | Yes | No | Hb-B |
| Malawi, 2016 | Ferritin | ELISA | RBP | ELISA | AAS | Immunoassay | Microbiologic assay | -- | Yes | Yes^2^ | ® 301^┼^ |
| Mexico, 2006 | Ferritin | UNK | -- | -- | ICP-OES | -- | -- | -- | Yes | Yes | UNK |
| Mexico, 2012 | Ferritin | UNK | -- | -- | -- | Immunoassay | Immunoassay | -- | Yes | Yes | UNK |
| Pakistan, 2011 | Ferritin | UNK | Retinol | UNK | AAS | Immunoassay | Immunoassay | Radioimmuno-assay | No | No | ® 201 |
| Papua New Guinea, 2005 | sTfR | UNK | RBP | UNK | -- | -- | -- | -- | Yes | Yes | ® 201 |
| United Kingdom, 2014 | Ferritin | UNK | Retinol | UNK | ICP-MS | Immunoassay | -- | HPLC | No | Yes | UNK |
| United States, 2006 | Ferritin | UNK | Retinol | UNK | -- | Radioassay | Radioassay | Radioimmuno-assay | No | Yes | ® 201^┼^ |
| Vietnam, 2010 | Ferritin | ELISA | Retinol | Reverse-phase HPLC | AAS | Microbiologic assay | Microbiologic assay | HPLC | No | No | ® 301 |

1. Serum transferring receptor: sTfR; retinol binding protein: RBP; atomic absorption spectrometer: AAS; inductively coupled plasma-optical emission spectrometry: ICP-OES; inductively coupled plasma-mass spectrometry: ICP-MS; unknown: UNK; micronutrient not in the survey indicated by a ‘--‘. ^┼^ represents venous blood collection for anemia, otherwise finger prick collection.
2. All respondents in Azerbaijan reported no smoking, only 1 respondent in Malawi reported smoking. ^*^ indicates venous blood collection for anemia assessment

Supplemental Table 3. Prevalence of undernutrition assessed using underweight and short stature, and the independence of multiple forms of undernutrition

| Geographic Grouping | Country, year | Underweight  (BMI < 18.5kg/m^2^) | Short stature  (height < 145cm) | MDI > 0 and underweight | Anemia and underweight | MDI > 0 and short stature | Anemia and short stature |
| --- | --- | --- | --- | --- | --- | --- | --- |
| Americas | Mexico, 2006 | 2.8 (1.8, 3.9) | 10.7 (8.8, 12.6) | 1.1 (0.6, 1.5) | 0.4 (0.1, 0.6) | 4.0 (3.0, 5.0) | 1.1 (0.6, 1.5) |
|  | Mexico, 2012 | 1.5 (0.9, 2.0) | 8.4 (7.3, 9.5) | 0.8 (0.4, 1.2) | 0.1 (0.0, 0.2) | 4.2 (3.4, 5.0) | 1.2 (0.8, 1.7) |
|  | Ecuador, 2012 | 1.8 (1.3, 2.3) | 9.5 (8.3, 10.7) | 1.1 (0.8, 1.5) | 0.2 (0.1, 0.4) | 5.9 (4.6, 7.2) | 1.9 (1.1, 2.7) |
|  | US, 2006 | 3.3 (2.5, 4.0) | 0.3 (0.1, 0.4) | 0.7 (0.4, 1.1) ^↓^ | 0.1 (0.0, 0.1) ^↓^ | 0.2 (0.0, 0.3) | -- |
|  | Colombia, 2010 | 5.5 (4.9, 6.2) | 2.9 (2.5, 3.4) | 1.6 (1.3, 2.0) | 0.5 (0.3, 0.6) | 0.7 (0.5, 0.9) | 0.2 (0.1, 0.3) |
| Europe /  E Mediterranean | Azerbaijan, 2013 | 4.8 (3.7, 6.0) | 1.1 (0.7, 1.4) | 3.1 (2.3, 4.0) | 1.8 (1.1, 2.5) ^↓^ | 0.7 (0.4, 1.1) | 0.3 (0.1, 0.5) |
|  | UK, 2014 | 2.2 (0.6, 3.9) | 0.02 (0.0, 0.1) | 1.6 (0.1, 3.1) | 0.03 (0.0, 0.1) | 0.02 (0.0, 0.1) | -- |
|  | Georgia, 2009 | 4.7 (3.5, 6.0) | 0.1 (0.0, 0.2) | 1.0 (0.4, 1.5) | 1.6 (0.9, 2.3) | -- | 0.1 (0.0, 0.2) |
|  | Afghanistan, 2013 | 5.2 (3.2, 7.3) | 3.4 (1.2, 5.7) | 5.0 (3.0, 7.0) | 4.9 (2.9, 8.9) | 3.1 (0.9, 5.3) | 0.7 (0.0, 1.4) |
|  | Pakistan, 2011 | 16.8 (15.8, 17.8) | 4.5 (4.0, 5.0) | 15.6 (14.6, 16.6) | 10.0 (9.2, 10.8) ^↑^ | 4.1 (3.6, 4.6) | 2.3 (1.9, 2.6) |
| Africa | Cameroon, 2009 | 8.6 (6.2, 11.1) | 0.6 (0.0, 1.2) | 8.0 (5.6, 10.4) | 4.1 (2.5, 5.8) | 0.4 (0.0, 0.9) | 0.2 (0.0, 0.5) |
|  | Côte d’Ivoire, 2007 | 9.0 (6.0, 12.0) | 0.7 (0.1, 1.2) | 7.1 (4.6, 9.5) | 4.9 (2.9, 6.9) | 0.5 (0.0, 0.9) | 0.6 (0.1, 1.0) |
|  | Malawi, 2016 | 8.7 (6.2, 11.2) | 2.8 (1.1, 4.6) | 6.4 (4.0, 8.8) | 2.2 (0.8, 3.6) | 2.0 (0.5, 3.6) | 0.3 (0.0, 0.9) |
| Southeast Asia / Western Pacific | Papua New Guinea, 2005 | 5.2 (3.1, 7.3) | 5.0 (2.6, 7.4) | 0.6 (0.0, 1.1) | 2.9 (1.2, 4.6) ^↑^ | 0.4 (0.0, 0.8) | 2.3 (1.0, 3.6) |
|  | Cambodia, 2014 | 13.3 (9.9, 16.7) | 6.5 (3.3, 9.7) | 9.7 (6.1, 13.2) | 7.8 (4.4, 11.1) | 4.6 (2.3, 6.9) | 3.3 (1.2, 5.4) |
|  | Laos, 2006 | 13.4 (10.0, 16.8) | 13.7 (9.0, 18.4) | 2.8 (1.3, 4.3) | 5.3 (3.6, 7.0) ^↑^ | 5.4 (2.9, 7.9) | 6.4 (3.2, 9.6) ^↑^ |
|  | Vietnam, 2010 | 20.1 (18.0, 22.3) | 6.9 (5.4, 8.4) | 15.2 (13.3, 17.2) | 2.3 (1.6, 3.1) | 5.9 (4.4, 7.3) ^↓^ | 1.4 (0.8, 2.1) ^↑^ |

To assess independence of undernutrition conditions, the modified Rao-Scott chi-square test was done for 85 comparisons: assessing 1 - underweight and short stature; 2 – MDI>0 and underweight; 3 – anemia and underweight; 4 – MDI>0 and short stature; 5 – anemia and short stature, all by survey. There were 10 statistically significant findings (p <0.05); ↑ indicates where the observed prevalence was higher than expected and ↓ indicates where the observed prevalence was lower than expected. Underweight and short stature prevalence not shown (Pakistan observed prevalence was higher than expected). MDI=micronutrient deficiency index, indicating any deficiencies. Smoking and altitude adjusted hemoglobin <12 g/dL defined as having anemia.

Supplemental Table 4. Adjusted odds ratios for DBM-MDI according to age, socioeconomic status, residence, and education for women of reproductive age survey, organized by geographic groupings: BRINDA project^1^

| Geographic Grouping | Country, year | Age 15-19  (ref=20-29)  aOR (95% CI) | Age 30-39 (ref=20-29)  aOR (95% CI) | Age 40-49 (ref=20-29)  aOR (95% CI) | Med SES  (ref = low SES)  aOR (95% CI) | High SES  (ref = low SES)  aOR (95% CI) | Urban  (ref = rural)  aOR (95% CI) | Education  (ref = low)  aOR (95% CI) |
| --- | --- | --- | --- | --- | --- | --- | --- | --- |
| Americas | Mexico, 2006 | 0.53 (0.33, 0.86) | 1.14 (0.79, 1.65) | 1.88 (1.27, 2.77) | 0.66 (0.48, 0.91) | 0.52 (0.31, 0.85) | 0.58 (0.42, 0.82) | 0.98 (0.71, 1.36) |
|  | Mexico, 2012 | -- | 1.67 (1.32, 2.11) | 1.89 (1.50, 2.39) | 0.75 (0.61, 0.93) | 0.69 (0.52, 0.92) | 1.00 (0.83, 1.21) | -- |
|  | Ecuador, 2012 | 0.60 (0.42, 0.85) | 1.80 (1.57, 2.06) | 2.47 (2.10, 2.90) | 1.05 (0.82, 1.33) | 1.05 (0.82, 1.33) | 1.27 (1.07, 1.51) | 0.99 (0.82, 1.19) |
|  | US, 2006 | 0.85 (0.63, 1.15) | 1.3 (0.98, 1.73) | 1.76 (1.33, 2.33) | 0.7 (0.55, 0.88) | 0.41 (0.31, 0.54) | -- | -- |
|  | Colombia, 2010 | 0.54 (0.33, 0.87) | 1.95 (1.34, 2.83) | 2.55 (1.79, 3.64) | 0.93 (0.67, 1.28) | 1.4 (0.94, 2.1) | 1.40 (0.98, 2) | 1.27 (0.99, 1.62) |
| Europe /  E Mediterranean | Azerbaijan, 2013 | 0.57 (0.36, 0.9) | 2.52 (1.98, 3.22) | 3.31 (2.56, 4.27) | 0.91 (0.74, 1.13) | 1.04 (0.78, 1.38) | 1.33 (1.05, 1.68) | 1.18 (0.73, 1.9) |
|  | UK, 2014 | 1.77 (0.35, 9.02) | 2.22 (1.08, 4.55) | 2.00 (1.01, 3.96) | 0.58 (0.33, 1.01) | 0.43 (0.25, 0.74) | -- | 0.79 (0.37, 1.72) |
|  | Georgia, 2009 | 0.48 (0.16, 1.49) | 2.02 (1.09, 3.74) | 3.45 (2.03, 5.85) | 2.07 (1.15, 3.74) | -- | 1.35 (0.84, 2.16) | 1.68 (0.7, 4.03) |
|  | Afghanistan, 2013 | 0.59 (0.19, 1.81) | 1.53 (0.78, 3.03) | 1.56 (0.39, 6.18) | 1.97 (0.77, 5.06) | 4.19 (1.75, 10.08) | -- | -- |
|  | Pakistan, 2011 | -- | 1.89 (1.68, 2.14) | 2.68 (2.22, 3.25) | 2.45 (2.13, 2.83) | 3.72 (3.07, 4.51) | 1.44 (1.26, 1.64) | 1.31 (1.14, 1.5) |
| Africa | Cameroon, 2009 | 0.36 (0.16, 0.81) | 1.53 (1.09, 2.16) | 2.82 (1.36, 5.86) | 2.00 (1.16, 3.45) | 3.14 (1.58, 6.24) | 1.17 (0.68, 2.02) | 1.40 (0.94, 2.09) |
|  | Côte d’Ivoire, 2007 | 0.16 (0.06, 0.44) | 1.41 (0.82, 2.41) | 2.22 (1.16, 4.27) | 1.75 (0.9, 3.38) | 2.04 (0.9, 4.62) | 1.84 (0.88, 3.85) | 1.54 (0.87, 2.71) |
|  | Malawi, 2016 | 0.51 (0.22, 1.2) | 1.02 (0.5, 2.09) | 2.24 (1.25, 4.01) | 2.39 (1.32, 4.34) | 2.48 (0.92, 6.65) | 2.67 (1.49, 4.79) | 2.00 (1.03, 3.86) |
| Southeast Asia / Western Pacific | PNG, 2005 | 0.42 (0.04, 4.66) | 2.66 (0.8, 8.77) | 0.93 (0.1, 8.75) | 1.06 (0.09, 13.16) | 13.52 (2.5, 73.14) | 0.46 (0.13, 1.62) | 2.43 (0.66, 8.98) |
|  | Cambodia, 2014 | -- | 1.83 (0.82, 4.06) | 2.26 (0.87, 5.88) | 2.12 (0.97, 4.60) | 4.52 (1.20, 170) | 0.88 (0.29, 2.67) | 0.68 (0.29, 1.6) |
|  | Laos, 2006 | ǂ | 1.19 (0.29, 4.91) | 0.67 (0.15, 3.06) | 1.60 (0.40, 6.39) | 1.82 (0.53, 6.22) | 0.77 (0.31, 1.86) | 0.86 (0.41, 1.81) |
|  | Vietnam, 2010 | ǂ | 1.97 (1.08, 3.58) | 3.50 (2.00, 6.12) | -- | -- | 0.85 (0.54, 1.34) | -- |

1. Models included all covariates (age, SES, urban/rural, and education) that were available per survey. For surveys with limitations in covariates, those not available to be included in multivariate models are indicated by a ‘--‘. ǂ in Cambodia and Laos, there were no DBM-MDI cases in the 15-19 age group. DBM-MDI: double burden of malnutrition-micronutrient deficiency index; BRINDA: Biomarkers Reflecting Inflammation and Nutritional Determinants of Anemia; Adjusted odds ratio: aOR; Confidence interval: CI; Reference: ref; Socioeconomic status: SES.

Supplemental Table 5. Adjusted odds ratios for DBM-anemia according to age, socioeconomic status, residence, and education for women of reproductive age by survey, organized by geographic groupings: BRINDA project^1^

| Geographic Grouping | Country, year | Age 15-19  (ref=20-29)  aOR (95% CI) | Age 30-39 (ref=20-29)  aOR (95% CI) | Age 40-49 (ref=20-29)  aOR (95% CI) | Med SES  (ref = low SES)  aOR (95% CI) | High SES  (ref = low SES)  aOR (95% CI) | Urban  (ref = rural)  aOR (95% CI) | Education  (ref = low)  aOR (95% CI) |
| --- | --- | --- | --- | --- | --- | --- | --- | --- |
| Americas | Mexico, 2006 | 0.33 (0.12, 0.96) | 0.79 (0.36, 1.74) | 1.16 (0.54, 2.48) | 0.90 (0.56, 1.44) | 0.37 (0.18, 0.73) | 1.41 (0.78, 2.56) | 1.09 (0.63, 1.92) |
|  | Mexico, 2012 | -- | 2.09 (1.43, 3.06) | 2.41 (1.65, 3.51) | 0.80 (0.57, 1.11) | 0.45 (0.3, 0.68) | 1.20 (0.88, 1.63) | -- |
|  | Ecuador, 2012 | 0.58 (0.24, 1.36) | 1.62 (1.27, 2.06) | 2.45 (1.85, 3.23) | 1.04 (0.74, 1.46) | 0.62 (0.4, 0.94) | 1.58 (1.25, 2.00) | 1.05 (0.74, 1.48) |
|  | US, 2006 | 1.26 (0.67, 2.36) | 1.70 (1.01, 2.85) | 3.02 (1.62, 5.64) | 0.55 (0.35, 0.85) | 0.28 (0.16, 0.48) | -- | -- |
|  | Colombia, 2010 | 0.07 (0.02, 0.35) | 1.83 (0.95, 3.53) | 3.18 (1.73, 5.85) | 0.75 (0.45, 1.24) | 0.66 (0.32, 1.34) | 1.26 (0.72, 2.21) | 1.32 (0.87, 1.99) |
| Europe /  E Mediterranean | Azerbaijan, 2013 | ǂ | 1.02 (0.80, 1.31) | 0.91 (0.70, 1.18) | 0.86 (0.30, 2.42) | 2.4 (0.82, 6.99) | 0.98 (0.65, 1.39) | 0.50 (0.14, 1.79) |
|  | UK, 2014 | 0.01 (0.00, 0.04) | 3.07 (0.31, 30.57) | 5.66 (0.65, 49.47) | 0.37 (0.12, 1.12) | 0.4 (0.12, 1.29) | -- | 1.16 (0.17, 7.74) |
|  | Georgia, 2009 | 0.77 (0.24, 2.50) | 2.41 (1.42, 4.07) | 4.71 (3.02, 7.35) | 1.40 (0.85, 2.30) | -- | 1.37 (0.84, 2.21) | 1.16 (0.59, 2.3) |
|  | Afghanistan, 2013 | 0.23 (0.02, 2.21) | 1.16 (0.48, 2.82) | 0.62 (0.12, 3.09) | 2.29 (0.84, 6.21) | 2.41 (0.88, 6.56) | -- | -- |
|  | Pakistan, 2011 | -- | 1.76 (1.51, 2.05) | 2.62 (2.07, 3.32) | 2.12 (1.75, 2.57) | 2.65 (2.08, 3.36) | 1.46 (1.24, 1.71) | 1.25 (1.05, 1.49) |
| Africa | Cameroon, 2009 | 0.81 (0.28, 2.33) | 1.10 (0.61, 1.97) | 1.36 (0.42, 4.45) | 2.84 (1.11, 7.28) | 5.21 (1.63, 16.59) | 1.79 (0.73, 4.37) | 0.80 (0.45, 1.39) |
|  | Côte d’Ivoire, 2007 | 0.42 (0.14, 1.24) | 1.71 (1.01, 2.89) | 1.73 (0.81, 3.69) | 0.8 (0.34, 1.90) | 1.04 (0.39, 2.77) | 1.83 (0.77, 4.33) | 1.66 (0.93, 2.97) |
|  | Malawi, 2016 | 0.15 (0.03, 0.75) | 0.5 (0.11, 2.14) | 1.93 (0.6, 6.18) | 4.99 (1.21, 20.54) | 1.98 (0.15, 26.02) | 2.54 (0.22, 29.65) | 1.33 (0.16, 11.12) |
| Southeast Asia / Western Pacific | Papua New Guinea, 2005 | 1.07 (0.27, 4.32) | 4.13 (1.80, 9.46) | 1.73 (0.63, 4.77) | 0.86 (0.32, 2.32) | 2.71 (1.00, 7.32) | 0.57 (0.20, 1.63) | 1.51 (0.67, 3.38) |
|  | Cambodia, 2014 | -- | 3.95 (1.19, 13.12) | 5.86 (1.63, 21.05) | 2.5 (1.03, 6.11) | 3.8 (0.53, 27.31) | 1.02 (0.23, 4.44) | 0.39 (0.13, 1.14) |
|  | Laos, 2006 | 0.46 (0.08, 2.70) | 2.41 (0.66, 8.82) | 2.11 (0.53, 8.43) | 6.41 (1.88, 21.87) | 9.62 (1.58, 58.41) | 1.17 (0.26, 5.28) | 0.5 (0.16, 1.56) |
|  | Vietnam, 2010 | 1.03 (0.83, 1.29) | 17.21 (14.3, 21.1) | 21.14 (18.1, 26.2) | -- | -- | 2.96 (0.69, 12.76) | -- |

1. Models included all covariates (age, SES, urban/rural, and education) that were available per survey. For surveys with limitations in covariates, those not included in multivariate models are indicated by a ‘--‘. ǂ in Azerbaijan, there were no DBM-anemia cases in the 15-19 age group. Double burden of malnutrition: DBM; Biomarkers Reflecting Inflammation and Nutritional Determinants of Anemia: BRINDA; Adjusted odds ratio: aOR; Confidence interval: CI; Reference: ref; Socioeconomic status: SES.

Supplemental Table 6. Adjusted odds ratios for predictors of overweight or obesity according to individual and household characteristics by survey, organized by geographic groupings: BRINDA project^1^

| Geographic Grouping | Country, year | Age 15-19  (ref=20-29)  aOR (95% CI) | Age 30-39 (ref=20-29)  aOR (95% CI) | Age 40-49 (ref=20-29)  aOR (95% CI) | Med SES  (ref = low SES)  aOR (95% CI) | High SES  (ref = low SES)  aOR (95% CI) | Urban  (ref = rural)  aOR (95% CI) | Education  (ref = low)  aOR (95% CI) |
| --- | --- | --- | --- | --- | --- | --- | --- | --- |
| Americas | Mexico, 2006 | 0.40 (0.27, 0.60) | 2.33 (1.59, 3.44) | 3.90 (2.61, 5.84) | 0.79 (0.59, 1.06) | 0.66 (0.39, 1.12) | 0..87 (0.62, 1.20) | 1.01 (0.74, 1.39 |
|  | Mexico, 2012 | -- | 2.78 (2.22, 3.49) | 4.14 (3.27, 5.24) | 0.91 (0.72, 1.14) | 0.73 (0.54, 1.00) | 1.31 (1.06, 1.62) | -- |
|  | Ecuador, 2012 | 0.48 (0.35, 0.64) | 2.36 (2.05, 2.72) | 3.74 (2.96, 4.72) | 1.2 (0.9, 1.6) | 0.9 (0.67, 1.21) | 1.03 (0.85, 1.25) | 0.81 (0.69, 0.96) |
|  | US, 2006 | 0.62 (0.51, 0.77) | 1.75 (1.36, 2.25) | 2.44 (1.86, 3.2) | 0.8 (0.6, 1.05) | 0.48 (0.38, 0.61) | -- | -- |
|  | Colombia, 2010 | 0.40 (0.31, 0.52) | 2.44 (1.92, 3.1) | 3.9 (3.03, 5) | 1.02 (0.81, 1.29) | 1.06 (0.76, 1.46) | 0.92 (0.72, 1.19) | 0.97 (0.81, 1.15) |
| Europe /  E Mediterranean | Azerbaijan, 2013 | 0.52 (0.36, 0.75) | 3.53 (2.69, 4.65) | 7.28 (5.57, 9.52) | 1.48 (1.19, 1.85) | 1.75 (1.27, 2.41) | 1.08 (0.83, 1.4) | 1.39 (0.85, 2.27) |
|  | UK, 2014 | 0.77 (0.18, 3.23) | 1.62 (0.9, 2.92) | 2.2 (1.21, 4) | 0.89 (0.49, 1.6) | 0.59 (0.35, 0.98) | -- | 0.59 (0.26, 1.32) |
|  | Georgia, 2009 | 0.52 (0.31, 0.88) | 2.36 (1.66, 3.37) | 7.11 (5.07, 9.96) | 1.21 (0.93, 1.58) | -- | 1.52 (1.13, 2.04) | 1.4 (0.82, 2.39) |
|  | Afghanistan, 2013 | 0.62 (0.22, 1.74) | 1.6 (0.82, 3.09) | 2.05 (0.8, 5.23) | 2.33 (0.93, 5.8) | 4.96 (2.04, 12.1) | -- | -- |
|  | Pakistan, 2011 |  | 2.67 (2.2, 3.23) | 2.51 (2.19, 2.89) | 3.88 (3.2, 4.71) | 1.42 (1.25, 1.62) | 1.32 (1.15, 1.52) | 2.67 (2.2, 3.23) |
| Africa | Cameroon, 2009 | 0.42 (0.2, 0.87) | 1.53 (1.09, 2.16) | 3.65 (1.66, 8.02) | 2.45 (1.41, 4.27) | 3.57 (1.84, 6.92) | 1.14 (0.68, 1.91) | 1.38 (0.94, 2.03 |
|  | Côte d’Ivoire, 2007 | 0.17 (0.07, 0.42) | 1.47 (0.9, 2.4) | 2.16 (1.15, 4.06) | 1.61 (0.86, 3.01) | 2.38 (1.11, 5.11) | 1.84 (0.94, 3.59) | 1.29 (0.74, 2.23) |
|  | Malawi, 2016 | 0.55 (0.24, 1.28) | 1.76 (0.91, 3.41) | 2.56 (1.43, 4.58) | 2.42 (1.4, 4.19) | 2.52 (1.02, 6.22) | 2.13 (1.26, 3.62) | 2.08 (1.17, 3.7) |
| Southeast Asia / Western Pacific | Papua New Guinea, 2005 | 2.73 (1.55, 4.84) | 2.15 (1.19, 3.88) | 1.67 (0.92, 3.02) | 0.75 (0.37, 1.5) | 0.98 (0.47, 2.07) | 2.64 (1.33, 5.25) | 1.37 (0.83, 2.29) |
|  | Cambodia, 2014 | -- | 1.79 (0.87, 3.67) | 2.81 (1.39, 5.69) | 2.08 (1.24, 3.49) | 2.5 (0.73, 8.53) | 1.45 (0.69, 3.04) | 0.92 (0.46, 1.85) |
|  | Laos, 2006 | 0.59 (0.21, 1.65) | 3.16 (1.31, 7.64) | 3.88 (1.64, 9.21) | 3.67 (1.9, 7.1) | 6.92 (2.79, 17.2) | 1.53 (0.73, 3.2) | 0.71 (0.39, 1.28) |
|  | Vietnam, 2010 | ǂ | 2.16 (1.21, 3.87) | 4.39 (2.55, 7.54) | -- | -- | 1.02 (0.67, 1.55) | -- |

1. Models included all covariates (age, SES, urban/rural, and education) that were available per survey. For surveys with limitations in covariates, those not included in multivariate models are indicated by a ‘--‘.ǂ in Vietnam, there were no OWOB cases in the 15-19 age group. Adjusted odds ratio: aOR; Biomarkers Reflecting Inflammation and Nutritional Determinants of Anemia: BRINDA; Confidence interval: CI; Double burden of malnutrition: DBM; Reference: ref; Socioeconomic status: SES.

Supplemental Table 7. Adjusted odds ratios for predictors of micronutrient deficiency > 0 (i.e., presence of at least one micronutrient deficiency) according to individual and household characteristics by survey, organized by geographic groupings: BRINDA project^1^

| Geographic Grouping | Country, year | Age 15-19  (ref=20-29)  aOR (95% CI) | Age 30-39 (ref=20-29)  aOR (95% CI) | Age 40-49 (ref=20-29)  aOR (95% CI) | Med SES  (ref = low SES)  aOR (95% CI) | High SES  (ref = low SES)  aOR (95% CI) | Urban  (ref = rural)  aOR (95% CI) | Education  (ref = low)  aOR (95% CI) |
| --- | --- | --- | --- | --- | --- | --- | --- | --- |
| Americas | Mexico, 2006 | 1.15 (0.79, 1.68) | 0.84 (0.60, 1.68) | 1.12 (0.78, 1.61) | 0.72 (0.53, 0.98) | 0.56 (0.36, 0.87) | 0.61 (0.45, 0.83) | 0.99 (0.74, 1.30) |
|  | Mexico, 2012 | -- | 0.94 (0.76, 1.16) | 0.93 (0.75, 1.16) | 0.76 (0.62, 0.93) | 0.75 (0.58, 0.98) | 0.82 (0.68, 0.98) | -- |
|  | Ecuador, 2012 | 1.12 (0.81, 1.54) | 1.03 (0.8, 1.32) | 1.23 (0.95, 1.59) | 0.86 (0.75, 0.99) | 1.05 (0.77, 1.44) | 1.46 (1.19, 1.8) | 1.18 (0.97, 1.43) |
|  | US, 2006 | 1.19 (0.95, 1.49) | 0.92 (0.7, 1.21) | 1.34 (1.07, 1.67) | 0.71 (0.56, 0.91) | 0.46 (0.36, 0.59) | -- | -- |
|  | Colombia, 2010 | 0.93 (0.71, 1.22) | 1.11 (0.86, 1.44) | 1.01 (0.78, 1.32) | 0.95 (0.74, 1.21) | 1.15 (0.83, 1.59) | 1.15 (0.89, 1.5) | 1.21 (1.01, 1.45) |
| Europe /  E Mediterranean | Azerbaijan, 2013 | 0.95 (0.7, 1.3) | 0.89 (0.7, 1.13) | 0.8 (0.62, 1.01) | 0.6 (0.48, 0.75) | 0.63 (0.46, 0.86) | 1.48 (1.15, 1.91) | 0.66 (0.41, 1.08) |
|  | UK, 2014 | 1.28 (0.28, 5.85) | 1.01 (0.51, 2) | 0.91 (0.49, 1.67) | 0.68 (0.39, 1.2) | 0.56 (0.33, 0.96) | -- | 1.31 (0.6, 2.83) |
|  | Georgia, 2009 | 0.5 (0.27, 0.93) | 1.2 (0.83, 1.73) | 1.39 (0.97, 2.01) | 1.58 (1.02, 2.46) | -- | 1.12 (0.76, 1.67) | 1.24 (0.65, 2.33) |
|  | Afghanistan, 2013 | 1.41 (0.25, 8.05) | 1.55 (0.71, 3.39) | 0.75 (0.18, 3.01) | 1.93 (0.43, 8.58) | 1.19 (0.38, 3.8) | -- | -- |
|  | Pakistan, 2011 |  | 0.98 (0.76, 1.27) | 0.94 (0.65, 1.34) | 1.2 (0.91, 1.58) | 1.35 (0.9, 2) | 1.17 (0.85, 1.63) | 0.65 (0.49, 0.85) |
| Africa | Cameroon, 2009 | 1.43 (0.47, 4.32) | 0.72 (0.41, 1.28) | 0.74 (0.25, 2.26) | 0.48 (0.24, 0.93) | 0.41 (0.17, 0.99) | 1.09 (0.56, 2.15) | 0.71 (0.4, 1.28) |
|  | Côte d’Ivoire, 2007 | 1.49 (0.67, 3.32) | 0.72 (0.4, 1.3) | 0.92 (0.48, 1.78) | 0.73 (0.46, 1.14) | 0.58 (0.27, 1.23) | 2.03 (1.16, 3.57) | 1.46 (0.78, 2.75) |
|  | Malawi, 2016 | 1.13 (0.61, 2.09) | 1.01 (0.54, 1.89) | 1.45 (0.69, 3.05) | 0.92 (0.56, 1.51) | 0.47 (0.24, 0.92) | 3.74 (1.12, 12.44) | 1.54 (0.91, 2.62) |
| Southeast Asia / Western Pacific | Papua New Guinea, 2005 | 1.09 (0.54, 2.21) | 0.7 (0.38, 1.3) | 0.86 (0.36, 2.03) | 1.25 (0.51, 3.1) | 4.03 (1.4, 11.56) | 0.3 (0.12, 0.8) | 1.09 (0.54, 2.21) |
|  | Cambodia, 2014 | -- | 0.89 (0.54, 1.45) | 0.63 (0.29, 1.39) | 0.53 (0.28, 1.03) | 0.78 (0.34, 1.76) | 1.18 (0.5, 2.79) | 0.89 (0.54, 1.45) |
|  | Laos, 2006 | 1.32 (0.88, 1.97) | 0.94 (0.56, 1.58) | 0.52 (0.29, 0.91) | 0.32 (0.22, 0.48) | 0.26 (0.12, 0.56) | 0.62 (0.31, 1.24) | 0.65 (0.37, 1.13) |
|  | Vietnam, 2010 | 0.91 (0.39, 2.08) | 0.87 (0.53, 1.41) | 0.79 (0.51, 1.23) | -- | -- | 0.77 (0.46, 1.27) | -- |

1. 1. Models included all covariates (age, SES, urban/rural, and education) that were available per survey. For surveys with limitations in covariates, those not included in multivariate models are indicated by a ‘--‘.Adjusted odds ratio: aOR; Biomarkers Reflecting Inflammation and Nutritional Determinants of Anemia: BRINDA; Confidence interval: CI; Double burden of malnutrition: DBM; Reference: ref; Socioeconomic status: SES.

Supplemental Table 8. Adjusted odds ratios for predictors of anemia according to individual and household characteristics by survey, organized by geographic groupings: BRINDA project^1^

| Geographic Grouping | Country, year | Age 15-19  (ref=20-29)  aOR (95% CI) | | Age 30-39 (ref=20-29)  aOR (95% CI) | | Age 40-49 (ref=20-29)  aOR (95% CI) | | Med SES  (ref = low SES)  aOR (95% CI) | | High SES  (ref = low SES)  aOR (95% CI) | | Urban  (ref = rural)  aOR (95% CI) | | Education  (ref = low)  aOR (95% CI) | |
| --- | --- | --- | --- | --- | --- | --- | --- | --- | --- | --- | --- | --- | --- | --- | --- |
| Americas | Mexico, 2006 | | 0.88 (0.45, 1.71) | | 0.90, 0.49, 1.66) | | 1.19 (0.65, 1.20) | | 0.91 (0.62, 1.33) | | 0.49 (0.29, 0.83) | | 1.30 (0.85, 2.00) | | 0.92 (0.62, 1.36) |
|  | Mexico, 2012 | | -- | | 1.58 (1.13, 2.2) | | 1.88 (1.34, 2.64) | | 0.83 (0.62, 1.11) | | 0.51 (0.35, 0.74) | | 1.14 (0.87, 1.49) | | -- |
|  | Ecuador, 2012 | | 0.91 (0.61, 1.34) | | 1.12 (0.94, 1.34) | | 1.52 (1.2, 1.94) | | 0.9 (0.72, 1.12) | | 0.54 (0.39, 0.74) | | 1.56 (1.27, 1.92) | | 0.95 (0.77, 1.18) |
|  | US, 2006 | | 1.68 (0.91, 3.12) | | 1.43 (0.83, 2.48) | | 2.62 (1.59, 4.3) | | 0.62 (0.45, 0.85) | | 0.35 (0.22, 0.54) | | -- | | -- |
|  | Colombia, 2010 | | 0.6 (0.37, 0.96) | | 1.05 (0.69, 1.61) | | 1.52 (1.01, 2.29) | | 0.71 (0.47, 1.06) | | 0.73 (0.42, 1.26) | | 1.19 (0.78, 1.83) | | 1.21 (0.89, 1.63) |
| Europe /  E Mediterranean | Azerbaijan, 2013 | | 0.82 (0.61, 1.12) | | 0.67 (0.53, 0.85) | | 0.7 (0.56, 0.88) | | 0.84 (0.65, 1.08) | | 0.78 (0.56, 1.09) | | 1.21 (0.89, 1.65) | | 1.17 (0.72, 1.9) |
|  | UK, 2014 | | 0.6 (0.09, 4.15) | | 1.59 (0.5, 5.07) | | 1.53 (0.55, 4.25) | | 0.56 (0.26, 1.23) | | 0.34 (0.15, 0.8) | | -- | | 2.6 (0.46, 14.72) |
|  | Georgia, 2009 | | 1.67 (1.02, 2.75) | | 1.55 (1.07, 2.24) | | 1.39 (1, 1.93) | | 1.17 (0.84, 1.63) | | -- | | 0.92 (0.65, 1.32) | | 0.81 (0.5, 1.32) |
|  | Afghanistan, 2013 | | 0.77 (0.2, 3.05) | | 1.35 (0.73, 2.5) | | 0.79 (0.42, 1.52) | | 0.97 (0.49, 1.93) | | 0.64 (0.36, 1.14) | | -- | | -- |
|  | Pakistan, 2011 | | -- | | 0.96 (0.87, 1.06) | | 1.1 (0.93, 1.29) | | 0.77 (0.68, 0.87) | | 0.64 (0.54, 0.77) | | 1.17 (1.03, 1.33) | | 0.92 (0.82, 1.04) |
| Africa | Cameroon, 2009 | | 0.87 (0.51, 1.48) | | 0.96 (0.67, 1.37) | | 0.66 (0.33, 1.31) | | 0.71 (0.44, 1.13) | | 0.77 (0.4, 1.48) | | 1.22 (0.75, 1.99) | | 0.77 (0.54, 1.1) |
|  | Côte d’Ivoire, 2007 | | 1.7 (0.95, 3.04) | | 1.08 (0.8, 1.46) | | 1.36 (0.88, 2.1) | | 0.72 (0.48, 1.09) | | 0.59 (0.34, 1.02) | | 0.96 (0.67, 1.36) | | 1.15 (0.73, 1.83) |
|  | Malawi, 2016 | | 1.8 (1.02, 3.16) | | 1.1 (0.58, 2.09) | | 1.87 (0.94, 3.72) | | 1.27 (0.76, 2.11) | | 0.9 (0.38, 2.1) | | 1.07 (0.35, 3.28) | | 1.25 (0.67, 2.32) |
| Southeast Asia / Western Pacific | Papua New Guinea, 2005 | | 0.93 (0.59, 1.49) | | 1.22 (0.78, 1.91) | | 1.11 (0.67, 1.84) | | 0.69 (0.33, 1.43) | | 1.15 (0.47, 2.83) | | 0.29 (0.11, 0.73) | | 1.12 (0.73, 1.73) |
|  | Cambodia, 2014 | | -- | | 1.62 (1.07, 2.46) | | 1.4 (0.6, 3.25) | | 1.21 (0.78, 1.9) | | 1.26 (0.52, 3.04) | | 1.12 (0.56, 2.24) | | 0.77 (0.42, 1.41) |
|  | Laos, 2006 | | 1.29 (0.8, 2.08) | | 1.04 (0.67, 1.61) | | 0.96 (0.55, 1.68) | | 0.54 (0.35, 0.83) | | 0.49 (0.25, 0.99) | | 0.96 (0.51, 1.81) | | 0.84 (0.57, 1.24) |
|  | Vietnam, 2010 | | 0.53 (0.27, 1.02) | | 1.15 (0.77, 1.7) | | 0.86 (0.58, 1.29) | | -- | | -- | | 0.93 (0.61, 1.41) | | -- |

1. Models included all covariates (age, SES, urban/rural, and education) that were available per survey. For surveys with limitations in covariates, those not included in multivariate models are indicated by a ‘--‘.Adjusted odds ratio: aOR; Biomarkers Reflecting Inflammation and Nutritional Determinants of Anemia: BRINDA; Confidence interval: CI; Double burden of malnutrition: DBM; Reference: ref; Socioeconomic status: SES.
